# Identification of novel genes and pathways in carotid atheroma using integrated bioinformatic methods

**DOI:** 10.1038/srep18764

**Published:** 2016-01-08

**Authors:** Wenqing Nai, Diane Threapleton, Jingbo Lu, Kewei Zhang, Hongyuan Wu, You Fu, Yuanyuan Wang, Zejin Ou, Lanlan Shan, Yan Ding, Yanlin Yu, Meng Dai

**Affiliations:** 1 Department of Health Management, Southern Medical University, Guangzhou 510515, Guangdong, China; 2 Department of Vascular Surgery, Nanfang Hospital, Southern Medical University, Guangzhou 510515, Guangdong, China; 3 Division of Epidemiology, School of Public Health and Primary Care, The Chinese University of Hong Kong, Hong Kong; 4 Laboratory of Cancer Biology and Genetics, National Cancer Institute, National Institutes of Health, 37 Convent Drive, Bethesda, MD 20892, USA; 5 Department of Vascular Surgery, People’s hospital of Henan province, Zhengzhou university, Zhengzhou 450003, Henan, China

## Abstract

Atherosclerosis is the primary cause of cardiovascular events and its molecular mechanism urgently needs to be clarified. In our study, atheromatous plaques (ATH) and macroscopically intact tissue (MIT) sampled from 32 patients were compared and an integrated series of bioinformatic microarray analyses were used to identify altered genes and pathways. Our work showed 816 genes were differentially expressed between ATH and MIT, including 443 that were up-regulated and 373 that were down-regulated in ATH tissues. GO functional-enrichment analysis for differentially expressed genes (DEGs) indicated that genes related to the “immune response” and “muscle contraction” were altered in ATHs. KEGG pathway-enrichment analysis showed that up-regulated DEGs were significantly enriched in the “FcεRI-mediated signaling pathway”, while down-regulated genes were significantly enriched in the “transforming growth factor-β signaling pathway”. Protein-protein interaction network and module analysis demonstrated that VAV1, SYK, LYN and PTPN6 may play critical roles in the network. Additionally, similar observations were seen in a validation study where SYK, LYN and PTPN6 were markedly elevated in ATH. All in all, identification of these genes and pathways not only provides new insights into the pathogenesis of atherosclerosis, but may also aid in the development of prognostic and therapeutic biomarkers for advanced atheroma.

Cardiovascular diseases are the leading cause of morbidity and mortality worldwide, and atherosclerosis is known to be the primary underlying factor responsible for the development of these diseases^1^. Despite extensive research, the detailed molecular mechanisms underlying the development of atherosclerosis and causing plaque rupture still remain unclear and new findings are urgently needed to complement the current knowledge and to identify new drug targets^2^. Rapid advances in biological technology, including DNA microarrays, able to detect the expression levels of tens of thousands of genes simultaneously, might help to provide comprehensive insights into the pathogenesis of atherosclerosis.

Gene-expression profiling of atherosclerosis has recently been used to identify genes and pathways relevant to vascular pathophysiology. It has previously been used to analyze altered gene expression in normal and diseased arteries^3^, establish crucial players in atherosclerotic plaque progression^4^,^5^, identify differentially expressed genes (DEGs) by comparing plaques with or without cerebrovascular symptoms^6^, discover candidate pathways and genes related to atherosclerosis^7^, and find gene expression changes of atherosclerotic plaques in different vascular beds^8^. However, some drawbacks are associated with those previous studies. In microarray studies comparing atheroma with normal tissues^3^,^7^, differences in the cellular compositions and morphologies of atherosclerotic plaques and normal arteries may result in differential gene expression profiles that simply reflect this variation^9^. In addition, irregular sample-collection methods existed in some studies^3^,^8^ , for example, samples from different sites or sources or small sample sizes, may affect the reliability of studies^10^. Furthermore, in animal model experiments^4^,^5^, a high degree of variability in plaque composition and gene expression between humans and animal models may limit the extension of cDNA array studies on animal material to clinical use^11^. Features of unstable plaques, such as surface ulceration, rupture, intraplaque hemorrhage and thrombus, may also occur in both asymptomatic and symptomatic patients, which may also confound studies^6^ that classify samples according to patient symptomatology^12^. Additionally, the relative lack of systematic bioinformatic analysis of cDNA microarrays in current studies limits the effective exploitation of gene-expression data sets^10^. Therefore, an integrated bioinformatic analysis based on cDNA microarray studies of human tissues may help to clarify the mechanisms underlying the development and progression of atherosclerosis.

To our knowledge, the variations between different individuals or blood vessels may affect the reliability of studies and it is very difficult to obtain healthy and diseased tissue from the same blood vessel of the same individual in human studies. To overcome the difficulty, we used a gene expression dataset from a previously published study^13^, comparing atheroma and its surrounding tissues from the same individual to track gene changes with disease progression and validated our findings with similar tissues. Besides, to interpret the biological relevance of these changes in gene expression, the microarray data were analyzed by integrated bioinformatic analysis expanding on traditional microarray analysis methods, namely gene-ontology and pathway analysis, thereby allowing the construction of interaction networks, that might identify novel prognostic markers and therapeutic targets.

## Results

### Identification of differentially expressed genes

**Figure 1:**
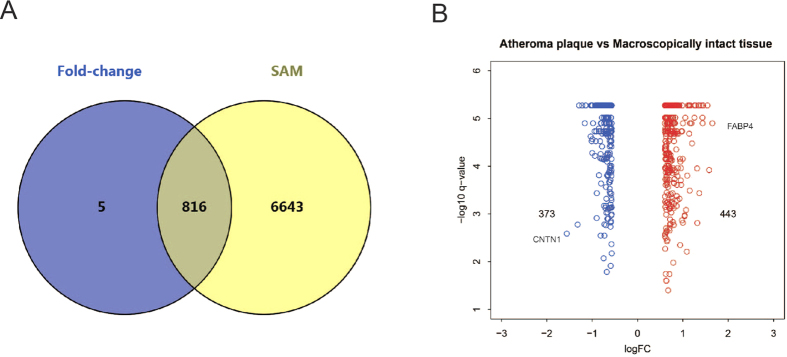
Differentially expressed genes were identified between ATH and MIT. (**A**) The overlapping gene set dually identified by the SAM and FC method. (**B**) Volcano plots for all differentially expressed genes in comparison. The dots indicate that up-(red) and down-regulated (blue) DEGs were significant both at false-discovery rate <0.05 and Fold-change >1.5 or <0.667.

Through our microarray analysis, a total of 816 differentially-expressed genes (DEGs) were identified between atheroma plaque (ATH) and macroscopically-intact tissue (MIT), including that 443 genes were up-regulated and 373 genes were down-regulated (Fig. 1A,B). The greatest fold differential expressions were a six-fold up-regulation of the FABP4 gene (fatty acid-binding protein 4) and a 3.3-fold down-regulation of the CNTN1 gene (contactin 1) in ATH compared with MIT.

### Gene ontology and pathway analyses

Two hundred-ninety and 26 GO terms were significantly enriched among the up-regulated and down-regulated genes, respectively. Table 1 shows the ten most overrepresented GO terms for the up-regulated and down-regulated DEGs, including immune-related biological process, such as “cell activation” and “cytokine production”, and “muscle system process”. Meanwhile, the KEGG pathway analysis identified 77 and 26 significantly enriched pathways for up-regulated and down-regulated genes, respectively. The ten most overrepresented KEGG pathways for up-regulated and down-regulated DEGs are shown in Table 2, with “B cell receptor signaling pathway” and “TGF-beta signaling pathway” being most significantly enriched.

### Protein–protein interaction (PPI) network analysis

**Figure 2:**
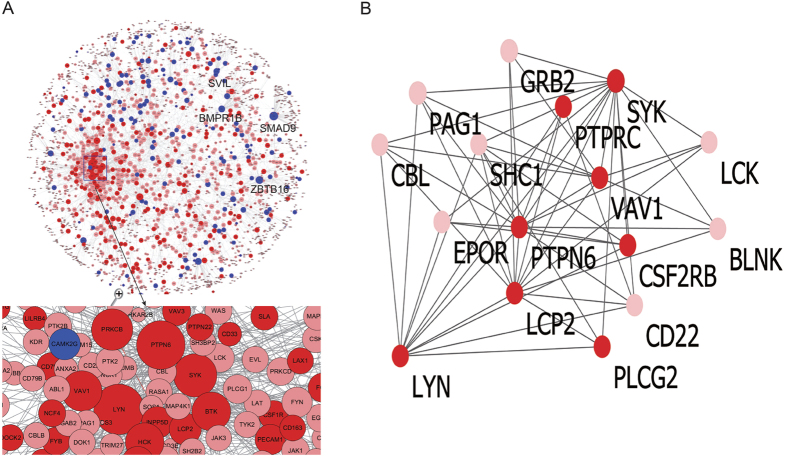
PPI interactions network of DEGs and the disease-relevant module found in the network. Nodes and links represent human proteins and protein interactions; Nodes represent the encoding genes of proteins; Red color indicates up-regulated genes annotated in the PPI network; Blue color indicates the down-regulated genes annotated in the PPI network; Pink nodes represent the non-DEGs which have an interaction with DEGs (**A**,**B**). The disease-relevant module contains the most nodes in CFinder software (**B**).

We constructed a PPI network to identify more important proteins and biological modules that may play crucial roles in the development of atherosclerosis. To confine the interactions only to those close to the DEGs, only first level interactions between DEGs and their neighbors were selected. There were 3,990 PPI pairs and 2,491 nodes in our constructed PPI network. The degree represents the number of neighboring nodes in the network and changes in the proteins/genes with higher degrees have more effects than changes in those with smaller degrees. SMAD9, LYN, PTPN6, ZBTB16, SYK, PRKCB, SVIL, VAV1, BMPR1B and BTK were located in the more important positions of network with higher degrees of 108, 102, 95, 81, 74, 66, 63, 62, 60 and 56, respectively, indicating those proteins play irreplaceable and critical roles in the maintaining the whole protein interactions in the network (Fig. 2A).

CFinder software was used to identify the disease-relevant modules in the PPI network. Figure 2B shows the module containing the most nodes with parameter k = 5. This module contained eight DEGs, including CSF2RB, LCP2, LYN, PLCG2, PTPN6, PTPRC, SYK and VAV1. Jointly using topology of network and module analysis to select candidates would identify genes that have higher significance in the PPI network. Finally, we focused on four genes (SYK, LYN, PTPN6 and VAV1) that were overlapped between the top 10 nodes ranked by degrees and the disease-relevant modules in the PPI network (Table 3).

### Verification of differentially expression genes in clinical samples

**Figure 3:**
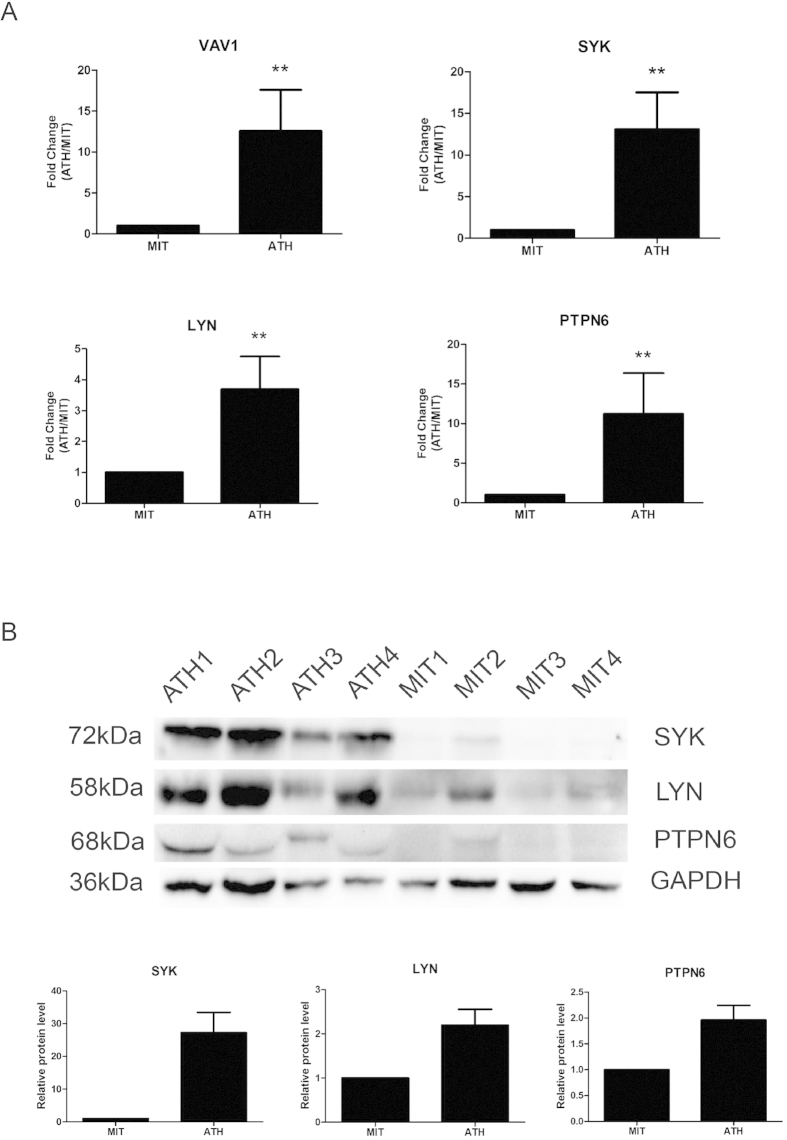
Validation of four candidate genes expression determined from microarray data analysis in clinical samples. (**A**) VAV1, LYN, SYK and PTPN6 expression was analyzed by qRT- PCR in 8 sets of atherosclerotic sample (8 ATH and 8 MIT, n = 16). *p < 0.05; **p < 0.01. The data is a representative of three independent experiments. (**B**) whole lysate from atherosclerotic samples (4 ATH and 4 MIT, n = 8) were analyzed by western blot. Top panel, the representative images of three independent experiments; Bottom panel, the quantitative data of the images in western blot by ImageJ software. The full-length blots are presented in Supplementary materials.

To confirm and validate the expression of four candidates determined from microarray data analysis in clinical samples, eight fresh sets of ATH and MIT samples were collected from surgery. The mRNA expression of four candidates (VAV1, SYK, LYN and PTPN6) were examined by qRT-PCR in eight sets of atherosclerotic sample (8 ATH and 8 MIT, n = 16). The results from qRT-PCR showed that mRNA level of VAV1 (12.6 ± 5.0), LYN (3.7 ± 1.1), SYK (13.1 ± 4.4) and PTPN6 (11.2 ± 4.9) increased by 12.6, 3.7, 13.1 and 11.2 folds respectively in ATH compared with in MIT (Fig. 3A). The results are consistent with data from microarray analysis although the differences in mRNA level were even higher than the differences determined in the microarray analysis. Moreover, the whole lysates from four sets of atherosclerotic samples (4 ATH and 4 MIT, n = 8) were analyzed by western blot. As Fig. 3B shows, the protein level of LYN (2.2 ± 0.4), SYK (27.3 ± 8.3) and PTPN6 (2.0 ± 0.5) significantly increased by degree of 2.2, 27.3 and 2.0 folds respectively in all ATH samples compared with MIT samples. We noticed that VAV1 was not detected in the atherosclerotic samples at the protein level by current antibody.

## Discussion

Microarray studies have great potential to provide novel insights into the pathogenesis of complex diseases. In the present study, we systematically applied integrated bioinformatic methods to mine new candidate players in the process of atherosclerosis and validated our findings in an independent set of samples at both mRNA and protein levels. In our study, we identified a total of 816 genes differentially expressed in ATH compared with MIT, including 443 up-regulated and 373 down-regulated DEGs. GO functional-enrichment analysis of these DEGs showed that genes mainly related to inflammation and immune responses were altered with disease progression. “Cell adhesion”, “proliferation”, “differentiation”, “motility”, “cell death”, “lipid metabolism” and “immune response” have all been reportedly associated with atherosclerosis^14^, and these processes were also identified in our enrichment results. Interestingly, although we identified similar numbers of up-regulated and down-regulated genes in ATHs, we observed an excess of significant GO categories for up-regulated genes, suggesting that the up-regulated genes are functionally more important in atherosclerosis progression.

Pathway-enrichment results showed an overabundance of immune and inflammatory signals, represented by the “chemokine-signaling pathway”, “natural killer cell-mediated cytotoxicity”, and “FcεRI-signaling pathway” in atherosclerosis. Our finding indicates that innate and adaptive immune cells might contribute to the development and progression of atherosclerosis, especially in the advanced stages. Hypercholesterolemia was initially considered to be the major risk factor for atherosclerosis, but recent advances have proven that chronic inflammation and autoimmunity play major roles in the initiation and progression of the disease^15^, as supported by our pathway-enrichment results. In addition, the “TGF-β signaling”, “calcium signaling”, and “osteoclast differentiation” were all significantly enriched among DEGs.

Mast cells are frequently in an activated state and participate in the process of atherosclerosis^16^. “FcεRI-mediated signaling” in mast cells is initiated by the interaction of an antigen with IgE bound to the extracellular domain of the α-chain of FcεRI. The mast cells activated by crosslinking of the FcεRI via IgE-antigen complexes could release and secrete biogenic amines, cytokines, lipid mediators and proteoglycans, which contribute to inflammatory responses. IgE and FcεRI have been implicated in several aspects of autoimmunity and chronic inflammatory diseases^17^. LYN, SYK and VAV1, the key modulators in our PPI network and module, are also implicated in this pathway (Figure S1). Our results indicated that this pathway might play a crucial role in the process of atherosclerosis; however, there is currently no proof of a relationship between “FcεRI-mediated signaling” and atherosclerosis. And it is worth noting that calcium signaling is associated with FcεRI -mediataed signaling. “Calcium signaling”^18^, “TGF-β signaling”^19^ and “osteoclast differentiation”^20^ have all been shown to be involved in the process of atherosclerosis. Our pathway-enrichment analysis supported the involvement of some pathways known to be associated with atherosclerosis initiation and progression, and also highlighted the FcεRI-mediated signaling pathway, which, to the best of our knowledge, has not previously been reported in association with carotid atheroma.

Our PPI network and further module analysis showed that VAV1, SYK, LYN and PTPN6 overlapped between the top 10 nodes and the disease-relevant module, suggesting that these genes play more crucial roles in the pathogenesis of carotid atheroma. Several studies have indicated important related functions for SYK and VAV1, suggesting that they play significant roles in atherosclerosis. SYK has been reported to be involved in the pathogenesis of atherosclerosis by activating monocyte chemotactic protein-1 expression^21^. Choi *et al*.^22^ found that TLR4/SYK-mediated macrophage responses may contribute to chronic inflammation in human atherosclerosis. Furthermore, the SYK inhibitor fostamatinib attenuated atherogenesis in mice, suggesting that SYK is a potential anti-inflammatory therapeutic target in atherosclerosis^23^. The validation study also indicated SYK was up-regulated both at the level of mRNA and protein which strengthens the assertion that it might play an important role in atherosclerosis development.

VAV1, a member of the VAV gene family, is expressed exclusively in hematopoietic cells. It is a signal transduction molecule that acts as guanine nucleotide exchange factor for Rac1 and Rho GTPases, and also functions as an adaptor platform^24^. VAV1 impacts on processes that are highly relevant to atherogenesis, such as NADPH oxidase-mediated generation of reactive oxygen species, cell death, and leukocyte activation. An *in vivo* carotid artery thrombosis model showed that genetic deletion of Vav1 and Vav3 together may prevent the development of occlusive thrombi in mice fed a high-fat diet^25^. Deletion of Vav1 alone led to modest inhibition of oxidized low-density lipoprotein uptake and foam-cell formation, while deletion of both Vav1 and Vav3 led to nearly complete inhibition of oxidized low-density lipoprotein uptake and foam-cell formation, suggesting that Vavs act as a critical regulator in the process of atherogenesis, and thus represents a novel therapeutic target^26^. In our validation study, VAV1 was not detected in the atherosclerotic samples at the protein level by current antibody, which worked well in the experiment of positive control tissues. Another VAV1 antibody was also applied, which still could not dectect the expression of this gene. Though we found similar expression patterns for upregulated VAV1 in both the qPCR and microarray analyses of ATH samples, VAV1 may not play a major role in the progression of atherosclerosis because its expression may be blocked at the translation phase.

LYN encodes a tyrosine protein kinase that is involved in the regulation of mast-cell degranulation. Lyn is the major Src-family kinase regulating glycoprotein VI signaling, and its absence caused a delay in activation and a marked reduction in platelet aggregation on collagen in a laser-injury model^27^. However, an apparently contradictory study showed that Lyn inhibited platelet activation, and that Lyn was increasingly inactivated as platelet aggregation progressed^28^. Miki *et al*.^29^ suggested that Lyn plays an important role in the metabolism of serum lipids, and could induce the expression of monocyte chemotactic protein-1, which is related to atherosclerosis, during the development of atherosclerotic lesions on high-fat diets. Previous studies reflect the complex roles of LYN and our validation experiments showed that LYN was up-regulated in the atheroma at the levels of mRNA and proteins, which might promote the progression of this disease.

PTPN6 is a member of the protein tyrosine phosphatase family of signaling molecules. It regulates a variety of cellular processes including cell growth, differentiation, mitotic cycle, and oncogenic transformation^30^. Kamata *et al*.^31^ suggested that PTPN6 acts as a negative regulator in the development of allergic responses such as allergic asthma. Dubois *et al*.^32^ concluded that PTPN6 played a crucial role in the negative modulation of insulin action and clearance in the liver, thus regulating whole-body glucose homeostasis. However, here we found PTPN6 up-regulated in atheroma at both the mRNA and proteins level and for the first time linked PTPN6 to atherosclerosis.

Additionally, to ensure the robustness of our candidate DEGs, we confirmed the expression of our candidate genes in another dataset GSE28829 (Supplementary Figure S2). The analysis revealed that a similar representation of the gene expression patterns of our candidate DEGs was seen in the dataset GSE28829, suggesting that VAV1, SYK, LYN and PTPN6 genes may play an important role in progression of atherosclerosis.

To our knowledge, this is the first integrated bioinformatic analysis comparing gene expression between carotid plaque and macroscopically intact arterial tissue. The large-scale gene expression profile analysis in our study is a significant strength in addition to the fact that paired tissue samples were obtained from the same individual. Our integrated methods are based on pre-specified specific algorithms, established topology information of networks and existing knowledge from databases and literature. The integrated methods have an advantage over traditional, single-analysis microarray approaches and other enrichment-analysis methods, such as DAVID^33^ and NetGestalt^34^ and ensure the reliability and accuracy of the results. Key candidates were also validated in Chinese patients, indicating the generalization of molecular mechanisms among different ethnic groups. Meanwhile, our integrated bioinformatic analysis might reveal the relationship between DEGs at molecular interaction and pathway levels, which provided some clues for the deeper mechanism of our candidate DEGs.

The discrepancies of expression between the qRT-PCR and microarray results may have been caused by a sensitivity bias between the two methods, difference in ethnicity, diet and lifestyle between French and Chinese people or by the use of different statistical methods in qRT-PCR and microarray. However, there are also some limitations in our study. First, the study population from the microarray analysis underwent carotid endarterectomy at the university hospital of Lyon, so the gene expression profile may be influenced by their ethnicity, diet and lifestyle. Secondly, the cohort consisted of older subjects that were predominantly male and the majority had hypertension. The generalization of our findings is unknown as the present results are limited to high risk populations with signs of atherosclerosis and severe symptoms. Finally, our work is a reanalysis of previously published dataset and although some previous work and our validation experiments support our gene expression analysis results, the work requires further study to identify the mechanism of action and to assess the relevance of our findings. This work serves as an excellent foundation and reference for further studies to expand on these findings in the future.

## Conclusion

This study identified SYK, LYN, PTPN6 and the “FcεRI-mediated signaling pathway” as potential candidate players involved in the pathogenesis of atherosclerosis. These findings enhance our understanding of the molecular mechanisms of this important disease. Further studies, such as gene functional studies, are needed to support the results of our study, with the aim of identifying candidate biomarkers with sufficient predictive power to act as prognostic and therapeutic biomarkers for advanced atheroma.

## Methods

### Source of data

An existing dataset GSE43292 within the Gene Expression Omnibus database was used for this work and obtained through approved access. The dataset was generated using the Affymetrix Human Gene 1.0 ST Array^13^. Ethical approval, sample tissue collection and preparation methods and characteristics of study participants were described in a previous report^13^. In brief, the dataset included 32 from 34 consecutive patients admitted to the university hospital of Lyon in 2009 for carotid endarterectomy. Paired samples were taken from individuals meaning that 64 carotid artery samples were analyzed. The mean age of participants was 70 years (±10 years) and the majority were male and with hypertension, with elevated blood lipid levels and just over one third of the sample were diabetic (Supplementary Table S1)^13^. Tissue samples were removed during surgery and dissected into two fragments: atheroma plaque tissue (ATH, subsequently identified as mostly stage IV and/or V lesions according to the American Heart Association classification) and macroscopically intact tissue (MIT, almost exclusively composed of stage I and II lesions)^13^.

### Identification of differentially expressed genes

The raw gene dataset obtained from the previous work^13^ was converted into expression measures, and background correction and quantile data normalization were performed using the robust multiarray average algorithm from the Affy package to obtain the expression profile data^35^. After deleting duplicated probes and averaging the multiple probes values for the same Entrez Genes (the unique integers as identifiers for gene records)^36^, we finally obtained expression profiles for 19,924 genes in the 64 samples.

Because the differentially expressed genes (DEGs) might have stronger relationship with the development of disease, the significance analysis of microarrays^37^ and fold-change methods were jointly used to identify DEGs between ATH and MIT.

### Functional-enrichment analysis

We integrated GO annotation into the total DEGs by mining for enriched GO terms of proteins using the R-based GO function software packages, which extracts biologically relevant terms from statistically significant GO terms for a disease^38^,^39^.

The DEGs were chosen for further analysis of Kyoto Encyclopedia of Gene and Genome (KEGG) enrichment. The SubpathwayMiner is a pathway identification system^40^ and accurately assessed the pathway structure to locate disease-relevant KEGG pathways^41^ and subpathways in DEGs relative to the genomic background.

### Protein–protein interaction network construction

In the study, we downloaded protein-protein interaction (PPI) data from human protein reference database (Release 9) on the website (http://www.hprd.org/)^42^. These interactions were derived from literature of experimental validation, including physical interactions and enzymatic reactions found in signal transduction pathways. The PPI data were preprocessed, including removing redundancy and self-loops, resulting in a connected network of 9,618 nodes (unique Entrez IDs) and 39,240 documented interactions. PPI networks are visualized in Cytoscape^43^ with the nodes representing the proteins/genes and the edges representing interactions between any two proteins/genes.

We constructed the PPI network by mapping the DEGs to the PPI network using the following steps. First, we extracted the nodes and relationships between DEGs and their direct interacting neighbors to confine the interactions only to those close to the DEGs using R software^44^, with each pair of interacting proteins in two lists of a text file. The DEGs (gene symbols) were listed in a NOA file with different node attribution annotations (down-regulated genes, up-regulated genes) and mapped to the constructed PPI network by the menu of “File-Import-Node Attributes”. Second, the degrees of nodes in the PPI networks were calculated by Network Analysis plugin by the menu of “Plugins- Network Analysis-Analyze network”. In our network, the degree of a node was the number of neighboring nodes in the network and node size was proportional to the degree of the protein. Third, CFinder software^45^ was used to find disease-related modules based on the Clique Percolation Method^46^, which is a free software for finding and visualizing overlapping dense groups of nodes in networks. PPI data from a text file was imported using the menu of “File- Open new network-run” with default parameters. The results of CFinder are highly correlated to the value of the parameter k. Larger k values correspond to smaller subgraphs with a higher density of links within them.

### Ethics statement and validation study sample collection

The collection of clinical samples was under approval by the Medical Ethics Committee of NanFang Hospital (Number: NFEC-2014-117) and informed consent was obtained from all subjects. The study was carried out in accordance with the standards set by the Declaration of Helsinki and Good Clinical Practice guidelines.

Human carotid atherosclerotic plaques were obtained from patients who underwent endarterectomy at the Vascular Surgery Department of Nanfang hospital of Southern medical university (Guangzhou, China). Patients (n = 8, mean age: 67.3 years, range: 53–80 years) with internal carotid artery stenosis >70% were included. The carotid atheromas were separated as ATH and MIT according to macroscopic observation. Dissected vascular tissues were rapidly frozen in liquid nitrogen and stored at –80 °C. Sample characteristics used for each experiment are shown in supplementary information Table S2.

### Quantitative real-time PCR (qRT-PCR)

Total RNA from specimens (n = 16) was isolated using Trizol reagent (TaKaRa Bio Inc, Japan) according to the manufacturer’s instructions. Complementary DNA was synthesized from 1000 ng of total RNA using the PrimeScript^TM^ RT reagent Kit with gDNA Eraser (TaKaRa Bio Inc, RR047Q) according to the manufacturer’s instruction, including the DNase step. Amplification was performed using SYBR^®^ Premix Ex Taq^TM^ (TaKaRa Bio Inc, RR420A). Quantitative real-time polymerase chain reaction (q-PCR) analysis was performed on Lightcycler96 (Roche Applied Science) according to the manufacturer’s protocol. GAPDH was used as internal control to normalize mRNA levels. All experiments were repeated three times. Primer sequences are listed in Table 4. Analysis was performed by the comparative delta–delta–Ct method^47^.

### Western blotting

Samples (n = 8) were taken from ultra-low temperature freezer and crushed in lysis buffer under liquid nitrogen. After detecting the concentration, proteins were separated on 7% SDS-polyacrylamide gel electrophoresis and transferred onto nitrocellulose membranes. Membranes were blocked with TBS-T(TBS/0.1% Tween-20) containing 5% non-fat dry milk for 1.5 hour at room temperature. Then, they were probed successively with mouse monoclonal anti-VAV1 (Cell Signaling Technology, Danvers, MA, USA), rabbit monoclonal anti-LYN (Abcam, Cambridge, MA, USA), rabbit monoclonal anti-PTPN6 (Abcam, Cambridge, MA, USA), and rabbit monoclonal anti-SYK (Abcam, Cambridge, MA, USA) antibodies at 4 °C overnight. Mouse monoclonal antibodies against GAPDH (Cell Signaling Technology, Danvers, MA, USA) were used as a loading control. Membranes were washed in TBS-T (TBS/0.1% Tween-20) three times for 5 min and probed with an anti-rabbit or -mouse HRP-conjugated secondary antibody in TBS-T with 5% of nonfat dry milk at room temperature for 1.5 h. Protein detection was performed using ECL reagents. Western blot bands were scanned using the ChemiDoc™ XRS Imaging System (BioRad, USA). Western blot bands were quantified using ImageJ software by measuring the band intensity for each group and normalized by GAPDH. The final results are expressed as fold changes by normalizing the data to the control values.

### Statistical analyses

Microarray analysis: DEGs for the microarray were identified using the fold-change and significance analysis of microarrays methods, with multiple testing corrections applied using the Benjamini-Hochberg method^48^. False-discovery rate <0.05 and fold-change >1.5 or <0.667 were set as the cutoff values of DEGs. For the functional enrichment analysis, significantly enriched GO terms in DEGs relative to the genomic background by GO function software packages were identified using the hypergeometric tests with an adjusted p-value <0.01, calculated by the Benjamini-Hochberg method^48^. Pathway-enrichment analysis was done using the R-based SubpathwayMiner software packages. Significantly enriched pathways were identified using hypergeometric tests and a p-value <0.01 was applied as the cut-off value for statistical significance.

Validation study: The data in this study are shown as the mean ± S.D. For the real time PCR, groups were compared using the Wilcoxon signed-rank test for continuous variables (SPSS 19.0, Chicago, IL) and a 2-sided p value <0.05 was considered statistically significant.

## Additional Information

**How to cite this article**: Nai, W. *et al*. Identification of novel genes and pathways in carotid atheroma using integrated bioinformatic methods. *Sci. Rep*. **6**, 18764; doi: 10.1038/srep18764 (2016).

## Supplementary Material

Supplementary Information

## Figures and Tables

**Table 1:** Top 10 most overrepresented GO terms for the DEGs.

Term	Name	Count	P-value	Adjustedp-value
Up-regulated DEGs				
GO:0001775	cell activation	83	0	0
GO:0001816	cytokine production	46	0	0
GO:0002250	adaptive immune response	33	0	0
GO:0002252	immune effector process	56	0	0
GO:0002253	activation of immune response	42	0	0
GO:0002376	immune system process	175	0	0
GO:0002443	leukocyte mediated immunity	33	0	0
GO:0002682	regulation of immune system process	104	0	0
GO:0002684	positive regulation of immune system process	76	0	0
GO:0002694	regulation of leukocyte activation	44	0	0
Down-regulated DEGs				
GO:0003012	muscle system process	32	3.33E-16	3.77E-11
GO:0006936	muscle contraction	28	6.35E-14	3.59E-09
GO:0003008	system process	73	1.08E-11	4.06E-07
GO:0032501	multicellular organismal process	157	2.01E-09	5.70E-05
GO:0007155	cell adhesion	46	8.28E-09	0.000167
GO:0022610	biological adhesion	46	8.87E-09	0.000167
GO:0048731	system development	106	1.95E-08	0.000314
GO:0044057	regulation of system process	28	2.64E-08	0.000373
GO:0007507	heart development	25	3.82E-08	0.000481
GO:0007399	nervous system development	64	1.30E-07	0.00147

Count: the number of differentially expressed genes; P-value was obtained by hypergeometric test; P-value was adjusted by Benjamini-Hochberg method. If the p-value is less than 2.2E-16 in R tool, it will be automatically changed to 0, and FDR should also be 0.

**Table 2:** Top 10 most overrepresented KEGG pathways for the DEGs.

Pathway id	Pathway name	Count	P-value	Adjustedp-value
Up-regulated DEGs				
path:04662	B cell receptor signaling pathway	14	0	0
path:04650	Natural killer cell mediated cytotoxicity	14	8.88178E-16	6.94556E-13
path:04664	Fc epsilon RI signaling pathway	11	2.87992E-13	9.65184E-11
path:04062	Chemokine signaling pathway	18	1.55209E-12	3.64121E-10
path:04380	Osteoclast differentiation	12	2.35412E-12	4.6023E-10
path:04666	Fc gamma R-mediated phagocytosis	10	5.50975E-11	8.07867E-09
path:04810	Regulation of actin cytoskeleton	12	1.0021E-09	1.17546E-07
path:05144	Malaria	6	1.90164E-08	1.71586E-06
path:04670	Leukocyte transendothelial migration	7	2.39578E-08	2.00732E-06
path:04610	Complement and coagulation cascades	8	7.67467E-08	5.00133E-06
Down-regulated DEGs				
path:04350	TGF-beta signaling pathway	4	0.0000639	0.075854499
path:04713	Circadian entrainment	5	0.000112157	0.075854499
path:04728	Dopaminergic synapse	6	0.000272488	0.109483429
path:04020	Calcium signaling pathway	8	0.000326677	0.109483429
path:04724	Glutamatergic synapse	6	0.00056299	0.146752804
path:04540	Gap junction	4	0.000822902	0.154822692
path:04723	Retrograde endocannabinoid signaling	3	0.000923945	0.154822692
path:04270	Vascular smooth muscle contraction	4	0.000934306	0.154822692
path:04730	Long-term depression	4	0.001055909	0.154822692
path:00380	Tryptophan metabolism	3	0.001142806	0.154862355

Count: the number of differentially expressed genes; P-value was obtained by hypergeometric test; P-value was adjusted by Benjamini-Hochberg method. If the p-value is less than 2.2E-16 in R tool, it will be automatically changed to 0, and FDR should also be 0.

**Table 3:** The candidate genes selected by our analysis.

symbol	entreze	FC	P-value	Ajusted P-value
SYK	6850	1.696016958	5.02E-07	5.38E-06
PTPN6	5777	1.694628747	5.02E-07	5.38E-06
LYN	4067	1.635340328	0*	0*
VAV1	7409	1.616980858	5.02E-07	5.38E-06

^*^If the p-value is less than 2.2E-16 in R tool, it will be automatically changed to 0, and FDR should also be 0.

**Table 4:** Primers for Real Time PCR.

Gene	Primer sequences (5′ → 3′)
VAV1	Forward primer: CAACCTGCGTGAGGTCAACReverse primer: ACCTTGCCAAAATCCTGCACA
SYK	Forward primer: TGCACTATCGCATCGACAAAGReverse primer: CATTTCCCTGTGTGCCGATTT
LYN	Forward primer: GCTTTTGGCACCAGGAAATAGCReverse primer: TCATGTCGCTGATACAGGGAA
PTPN6	Forward primer: TGAACTGCTCCGATCCCACTAReverse primer: CACGCACAAGAAACGTCCAG
GAPDH	Forward primer: GATGACATCAAGAAGGTGGTGAReverse primer: GTCTACATGGCAACTGTGAGGA
