# Corrigendum: Identification of novel genes and pathways in carotid atheroma using integrated bioinformatic methods

**DOI:** 10.1038/srep30366

**Published:** 2016-08-19

**Authors:** Wenqing Nai, Diane Threapleton, Jingbo Lu, Kewei Zhang, Hongyuan Wu, You Fu, Yuanyuan Wang, Zejin Ou, Lanlan Shan, Yan Ding, Yanlin Yu, Meng Dai

Scientific Reports 6: Article number: 1876410.1038/srep18764; published online: 01 08 2016; updated: 08 19 2016.

In the original version of this Article, there was an error in Affiliation 1 which was incorrectly given as ‘Department of Health Management, Southern Medical University, Guangzhou 510515, Guangdong, China’. The correct affiliation is listed below:

‘Department of Health Management, Nanfang Hospital, Southern Medical University, Guangzhou 510515, Guangdong, China’.

This error has now been corrected in the PDF and HTML versions of the Article.

