# Supplementary material for: Identification of novel genes and pathways in carotid atheroma using integrated bioinformatic methods
Source: Sci Rep. 2016 Jan 8;6:18764. doi: 10.1038/srep18764 (PMC4705461; doi:10.1038/srep18764)
Supplement: Supplementary Information [file srep18764-s1.pdf]

Identification of novel genes and pathways in carotid atheroma using integrated bioinformatic methods

Wenqing Nai[1*], Diane Threapleton[3*], Jingbo Lu[2*], Kewei Zhang[5], Hongyuan Wu[1], You Fu[1], Yuanyuan Wang[1], Zejin Ou[1], Lanlan Shan[1], Yan Ding[1], Yanlin Yu[4], Meng Dai[1]

[1]Department of Health Management, [2]Department of Vascular, Nanfang Hospital, Southern Medical University, Guangzhou 510515, Guangdong, China, [3]Division of Epidemiology, School of Public Health and Primary Care, The Chinese University of Hong Kong, Hong Kong, [4]Laboratory of Cancer Biology and Genetics, National Cancer Institute, National Institutes of Health, 37 Convent Drive, Bethesda, MD 20892, USA, [5] Department of Vascular, People's hospital of Henan province, Zhengzhou university, Zhengzhou 450003, Henan, China.

***These authors contributed equally to this work**

Correspondence and requests for materials should be addressed to M.D. (dm42298@126.com) or Y.L.Y. (yuy@mail.nih.gov) (Tel: +86 020 61642298; Fax: +86 020 62786002)

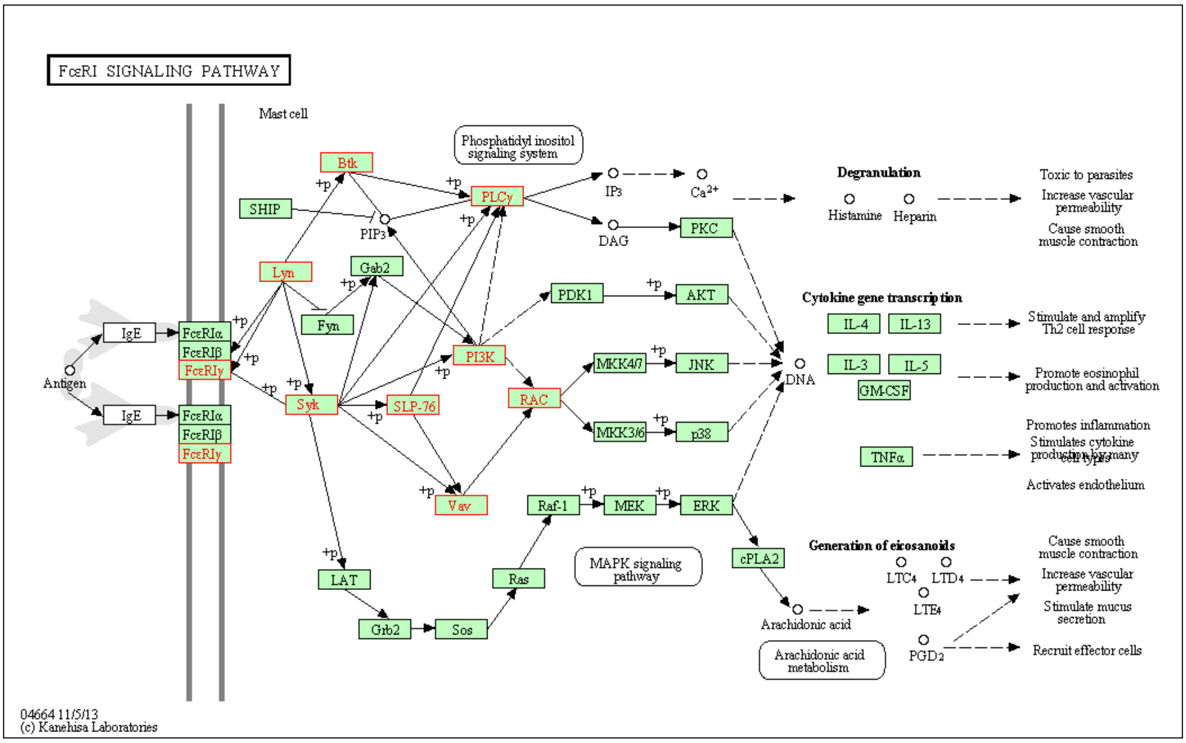

Supplementary Figure S1 FcεRI-mediated signaling where the differential genes were annotated. Enzymes (rectangular nodes) mapped by differential genes are shown with red node labels and borders.

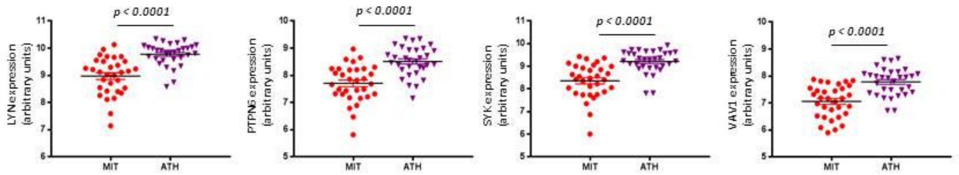

Expression of candidate genes in study of GSE43292 dataset with 32 atheroma plaque (ATH) and 32 distant macroscopically intact tissues (MIT). The data analyses were performed by two-tailed paired t test using GraphPad Prism 6 software.

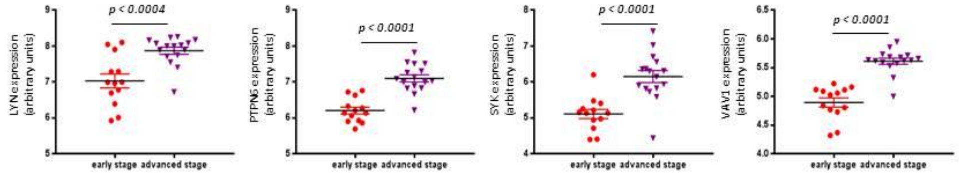

Expression of candidate genes in study of GSE28829 dataset with 13 early atherosclerotic plaque lesions (early stage) and 16 advanced atherosclerotic plaque lesions (advanced stage). The data analyses were performed by two-tailed unpaired t test using GraphPad Prism 6 software.

Supplementary Figure S2 Expression of candidate genes in two datasets (GSE43292 and GSE28829).

Supplementary Table S1

Clinical parameters from patients included in microarray analysis [1]

Clinical parameters	Clinical parameters
**Age, years**	70±10
**Gender**	5/29
**Hypertensive**	76%
**Diabetic,% 38**	38%
**Symptomatic**	40 %
**Lipidemic**	70%
**BMI, kg/m^2**	26±4
**SBP, mmHg**	156±23
**DBP, mmHg**	83±11
**Plasma glucose, mmol/L**	6±1
**LDL, mmol/L**	2.7±1
**HDL, mmol/L**	1±0.3
**Triglycerides, mmol/L**	1.8±1
**ApoB, g/L**	0.9±0.2
**HbA1c, %**	6.5±1%
**CRP, mg/L**	16±24
**Statin,**	62%
**ACEI/ARB,**	50%

Results are the mean ± SD. BMI indicates body mass index, SBP : systolic blood pressure, DBP : diastolic blood pressure, LDL : lowdensity lipoprotein, HDL : high-density lipoprotein, ApoB : apolipoprotein B, HbA1c : glycosylated hemoglobin, CRP : C-reactive protein. ACEI/ARB : angiotensin I converting enzyme inhibitors/ angiotensin II receptor blockers.

Supplementary Table S2

Clinical parameters from 8 patients included in validation study

Clinical parameters	Clinical parameters
**Age, years**	67.3±8.0
**Gender F/M**	3/7
**Hypertensive**	70%
**Diabetic**	50%
**Symptomatic**	60%
**Lipidemic**	80%
**BMI, kg/m^2**	22±3.6
**SBP, mmHg**	159±32
**DBP, mmHg**	83.6±11
**Plasma glucose, mmol/L**	7.8±5.4
**LDL, mmol/L**	3.0±1.1
**HDL, mmol/L**	1.0±0.2
**Triglycerides, mmol/L**	1.6±0.6
**ApoB, g/L**	1.3±0.4
**HbA1c, %**	6.6±1.0%
**CRP, mg/L**	17.3±26.7
**Statin**	50%
**ACEI/ARB**	40%

Results are the mean ± SD. BMI indicates body mass index, SBP : systolic blood pressure, DBP : diastolic blood pressure, LDL : lowdensity lipoprotein, HDL : high-density lipoprotein, ApoB : apolipoprotein B, HbA1c : glycosylated hemoglobin, CRP : C-reactive protein. ACEI/ARB : angiotensin I converting enzyme inhibitors/ angiotensin II receptor blockers.

Reference

1. Ayari, H. & Bricca, G. Identification of two genes potentially associated in iron-heme homeostasis in human carotid plaque using microarray analysis. *J Biosci* **38**, 311-315 (2013).

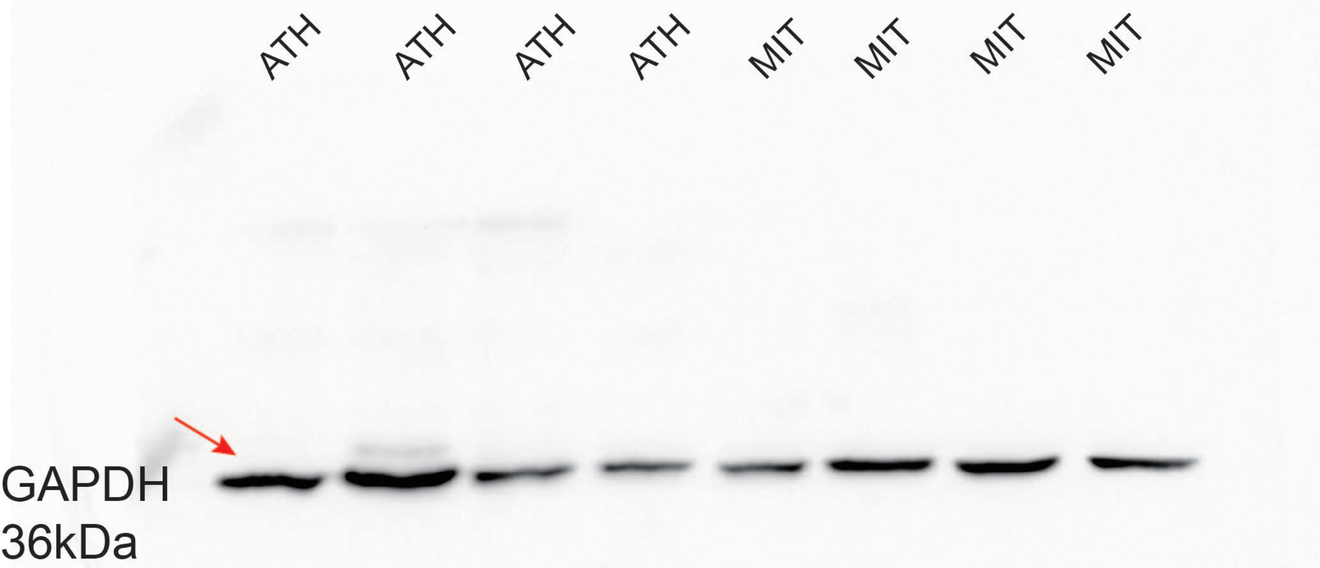
ATH
ATH
ATH
ATH
MIT
MIT
MIT
MIT
GAPDH
36kDa

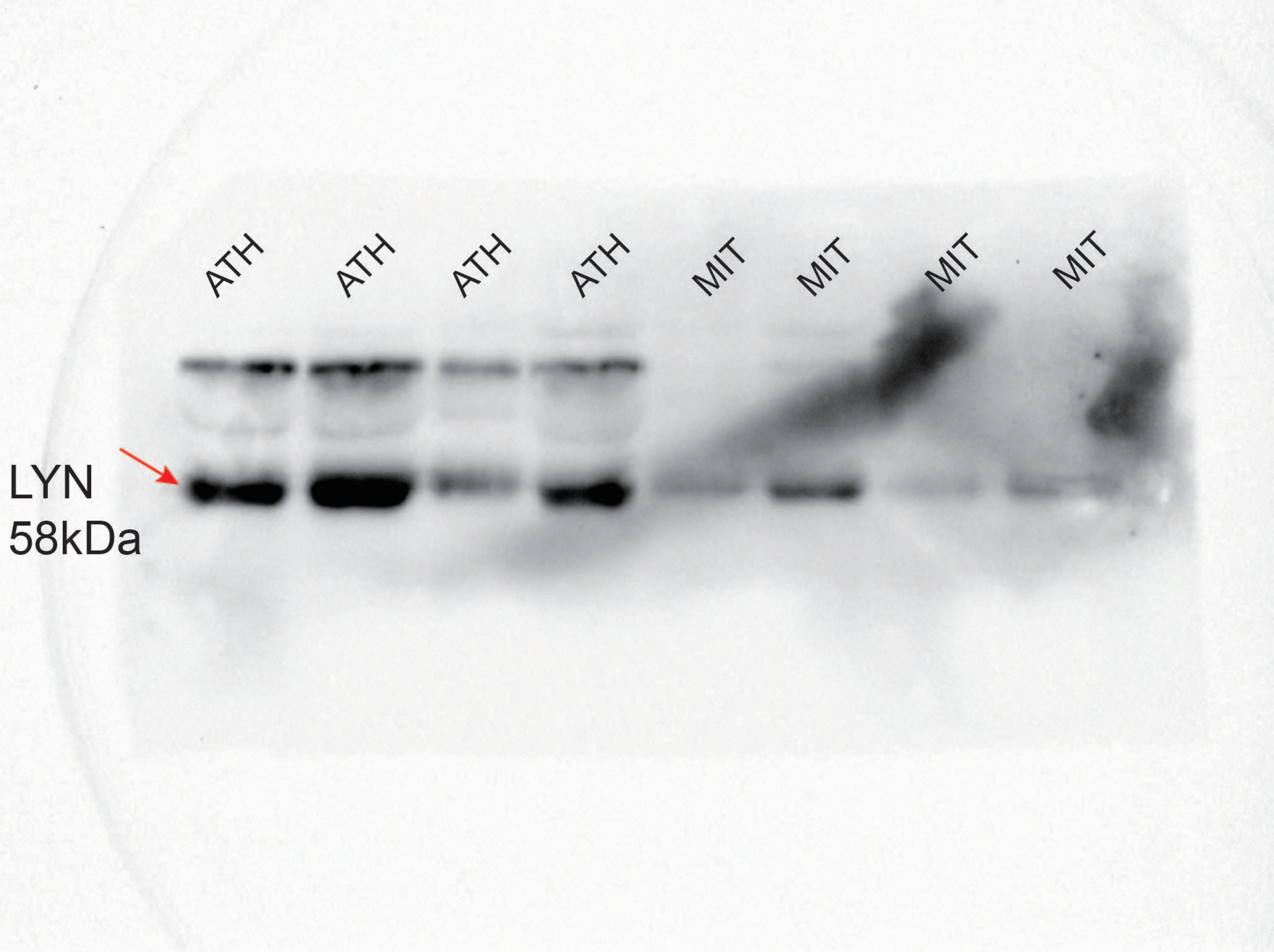
ATH
ATH
ATH
ATH
MIT
MIT
MIT
MIT
LYN
58kDa

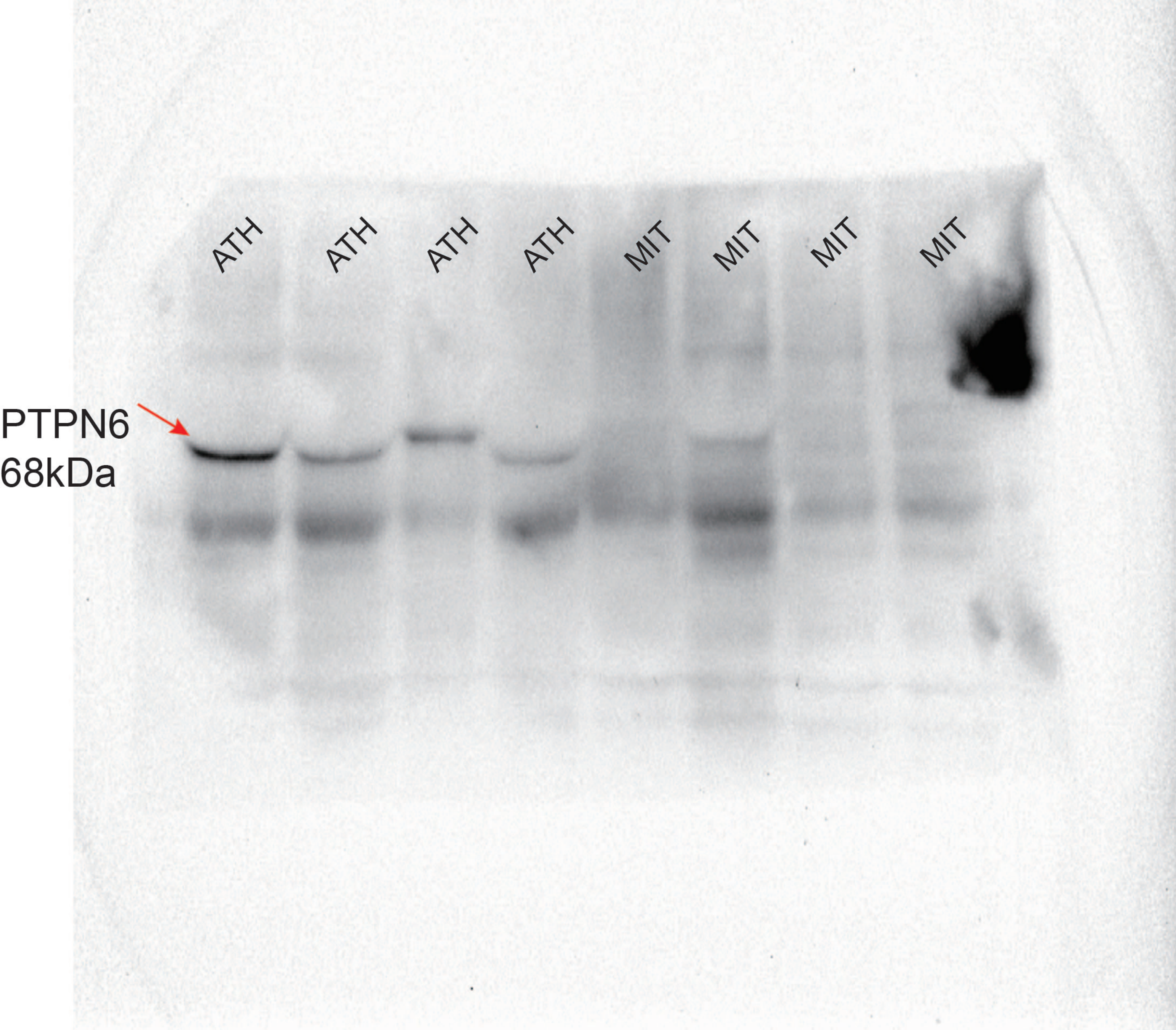
ATH
ATH
ATH
ATH
MIT
MIT
MIT
MIT
PTPN6
68kDa

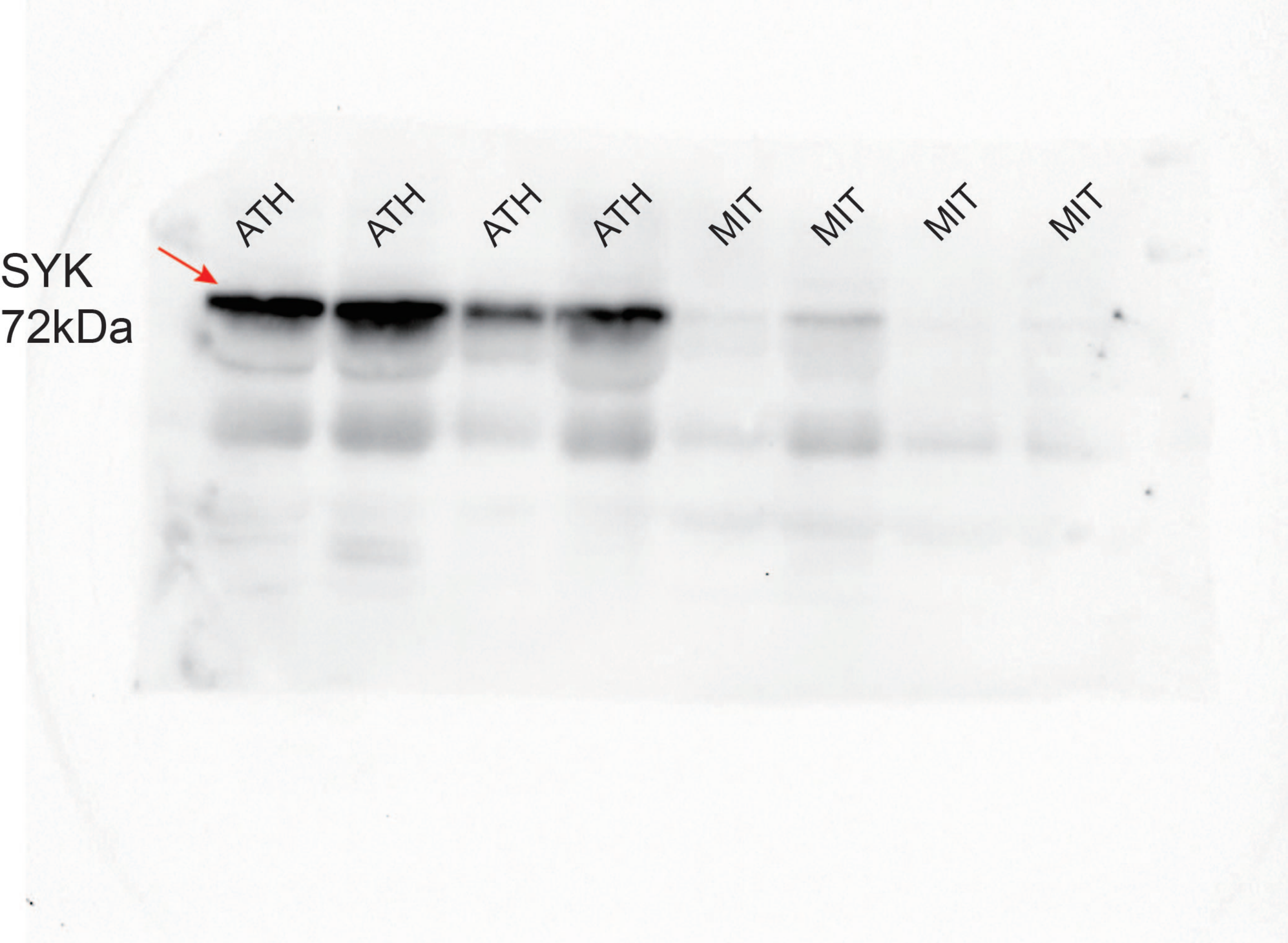
SYK
72kDa
ATH
ATH
ATH
ATH
MIT
MIT
MIT
MIT
